# Rethinking suicide related searches and their association with suicide rates, attempts, and self harm hospitalisation

**DOI:** 10.1038/s41746-025-01916-4

**Published:** 2025-08-29

**Authors:** Sandersan Onie, Matthew J. Coleshill, Michelle Torok, Venkatesha, Fiona Shand, Mark E. Larsen

**Affiliations:** 1 Black Dog Institute, University of New South Wales, Sydney, NSW Australia; 2 Wellspring Center Indonesia, Jakarta, Jakarta Selatan Indonesia; 3 UNSW Medicine, University of New South Wales, Sydney, NSW Australia; 4 Mark Wainwright Analytical Centre, University of New South Wales, Sydney, NSW Australia; 5 Centre for Big Data Research in Health, University of New South Wales, Sydney, NSW Australia

**Keywords:** Psychology, Disease prevention, Health policy, Health services, Epidemiology

## Abstract

This study explored the relationship between Google search volumes for suicide-related keywords and suicide, attempt, and self-harm hospitalisation rates across three investigations in Indonesia and Australia. While past research suggested a link between suicide-related searches and suicide rates, this study found no consistent association with suicide rates. However, search volumes were positively associated with suicide attempts in Indonesia and self-harm hospitalisations in Australia. Further analysis using the IMV-Model of Suicidal Behaviour showed that searches related to distress were not associated with outcomes, while ideation-related keywords were linked only to attempts. In contrast, searches related to specific suicide methods—reflecting the volitional phase of the IMV-Model—were associated with both attempts and suicides, particularly for high-lethality methods. Limitations include data quality on suicide outcomes, the representativeness of Google Trends, and a focus on only two countries. Overall, search behaviour may reflect attempt risk more than suicide risk, depending on keyword category.

## Introduction

Previous research has identified a link between suicide rates and Google searches for suicide-related keywords. These studies typically are epidemiological in nature, investigating the association between relative search volumes extracted using Google Trends, commonly for ‘suicide’ and ‘suicide methods’ keywords, and suicide rates across a period of time or across geographic regions^1–12^. These findings indicate that tracking internet search trends could offer a rapid and cost-effective method for monitoring trends in suicidal behaviour. However, conflicting evidence exists, with other studies failing to find this association^13–15^. In particular, a reanalysis of previous studies examining the association between suicide keywords and suicide rate observed mixed findings between countries and concluded that Google Trends data were unreliable in predicting suicide rate^14^. Such disparate findings are perhaps unsurprising due to how factors affecting the transition from ideation to an attempt, and the fatality of the attempt, vary both geographically and temporally. For example, methods used for suicide may vary across urban and rural locations due to the accessibility of different means. As a result, this may obscure the statistical relationship at a macro level. Despite initial enthusiasm for using Google Trends to predict suicide rates, these contradictory findings have raised doubts about its reliability.

While most studies focus on suicide rates, very few studies have investigated the relationship between search volumes and self-harm and suicide attempts, and how these keywords map onto existing models of suicidal behaviour. The Integrated-Motivational Volitional Model of Suicidal Behaviour (IMV-Model)^16^ suggests that there are three distinct phases to suicide: a pre-motivational phase with background and triggering events; a motivational phase in which the individual experiences defeat and entrapment, and subsequently a suicidal ideation and intent subphase; followed by a volitional phase in which an attempt is made. For every individual, myriad factors may influence whether they transition from ideation to attempt, as well as whether that attempt is fatal. The model, however, does not outline factors pertaining to the fatality of these attempts. Given that internet searches could occur throughout the motivational phase (during distress and ideation), which are more proximal to an attempt than to a suicide death, one hypothesis is that Google searches and self-harm and attempt rates may have a more robust statistical relationship.

Across three studies, we investigated the relationship between Google searches and rates of suicidal behaviours in Indonesia and Australia. In the first study, we assessed the relationship between provincial relative search volumes for suicide-related keywords with suicide rate and attempt rate on a geographic dimension in Indonesia for 2021. In the second study, we conceptually replicated the first study by assessing the relationship between national relative search volumes with suicide deaths and hospitalisations due to self-harm in Australia between 2008 and 2020. In the third study, we investigated whether keywords mapping onto phases of the IMV-Model are differentially associated with attempt/self-harm and suicide rate.

Australia and Indonesia were selected for this study because both countries differ in population composition, income level, and suicide prevention activities. Both countries provided reliable access to suicide and self-harm data for the relevant geographic regions (Study 1 and Study 3) and time periods (Study 2), and the research team had linguistic expertise in the languages spoken in these countries, enabling accurate keyword selection for Google Trends analysis. Furthermore, a vast majority of studies in this field examine high-income countries. Indonesia was a critical country to examine, given it is not a high-income country, and one of the most populous countries.

## Results

### Study 1

The analyses showed no evidence of a relationship between provincial suicide and suicide attempt rates (*r* = 0.031, *p* = 0.862).

There was mixed evidence of a relationship between provincial relative search volumes and suicide rates, with only ‘gantung diri’ (hang myself) demonstrating a significant positive correlation with suicide rates (*r* = 0.420, *p* = .008). However, there was a significant positive relationship between the provincial suicide attempt rates and the relative search volumes for ‘bunuh diri’ (suicide; *r* = 0.333, *p* = .027), ‘cara bunuh diri’ (suicide methods; *r* = 0.649, *p* < .001), the combined index (*r* = 0.506, *p* = .001), and ‘gantung diri’ (hang myself; *r* = 0.375, *p* = 0.017). Please see Table 1 for Study 1 analysis results.

**Table. 1:** Correlation of relative search volumes with suicide and attempt rate (at the provincial level in Indonesia, 2021)

Keyword	Correlation Coefficient	
	Suicide Rate	Attempt Rate
Bunuh Diri (Suicide, commit suicide)	0.189	0.333*
Cara Bunuh Diri (How to suicide/ how to kill yourself/ suicide methods)	-0.116	0.649***
Combined	0.021	0.506**
Gantung diri (Hang myself)	0.420**	0.375*

Note: * indicates *p* < 0.05, ** indicates *p* <.01, *** indicates p < .001

The cross-correlation analysis showed no evidence of a relationship between yearly suicide and hospitalisation for self-harm rates (*r* = −0.303; not significant).

### Study 2

There was no evidence of a relationship between suicide rate and the yearly relative search volumes for any of the individual or combined keywords (see Table 2). However, there was a significant positive relationship between the yearly self-harm hospitalisation rates and the relative search volumes for ‘suicide’ (*r* = 0.596), ‘how to suicide’ (*r* = 0.801), and the combined index that excluded painless suicide (0.600; see Table 2).

**Table. 2:** Correlation of relative search volumes with suicide rate and self-harm hospitalisation rate (2008-2020)

Keyword	Cross-Correlation Coefficient	
	Suicide Rate	Self-Harm Hospitalisation Rate
Suicide	−0.129	0.596^a^
How to commit suicide	0.217	0.038
How to suicide	−0.408	0.801^a^
How to kill yourself	−0.154	0.389
Painless suicide	−0.156	0.122
Commit Suicide	0.487	−0.188
Combined	−0.254	0.500
Combined (excluding ‘Painless suicide’)	0.259	0.600^a^

^a^Indicates a significant cross-correlation coefficient at time 0 indicated by when the cross-correlation coefficient exceeds the 95% confidence interval around the null.

### Study 3

The aggregated search volumes for the distress and ideation keyword groups were significantly correlated (*r* = 0.451, *p* = 0.004), therefore their respective correlations with suicide and attempt rates controlled for each other. Neither the distress group or ideation group correlated with the methods group (*r* = 0.179, *p* = 0.155; *r* = 0.207, *p* = 0.120; respectively).

The main analyses revealed that distress keywords, whether combined or individually, did not show a significant positive relationship with attempt or suicide rate (see Table 3). As was already shown in Study 1, the individual ‘bunuh diri’ and ‘cara bunuh diri’ ideation keywords showed a relationship with attempt rate, but not suicide rate. The same pattern was observed for the combined ideation group, despite the addition of the ‘ingin mati’ keyword, which did not correlate with either attempt or suicide rate. The combined methods keyword group showed a relationship with both suicide and attempt rate. However, there is a mixed pattern with individual keywords showing a relationship with both attempt and suicide, only one outcome, or neither. The results of the correlation analyses can be found in Table 3.

**Table. 3:** Correlation of the relative search volumes of IMV category keywords with attempt and suicide rates in Indonesia 2021

Keyword	Correlation Coefficient	
Groups	Suicide Rate	Attempt Rate
Distress (controlling for Ideation)	0.140	−0.189
Ideation (controlling for Distress)	−0.061	0.462**
Methods	0.317*	0.435**
**Distress**		
Depresi (Depression)	0.108	−0.077
Sedih banget (Depression)	−0.370	−0.024
Stress (Stress)	0.216	−0.231
Menyerah (Giving up)	0.367	0.132
Menyerah saja (Giving up)	0.286	0.17
Cape (Fatigue and burnout)	−0.018	−0.289
Lelah (Fatigue and burnout)	0.011	0.157
Aku lelah (Fatigue and burnout)	−0.120	0.185
**Ideation**		
Bunuh diri (Suicide, commit suicide)†	0.189	0.333*
Cara bunuh diri (How to suicide, how to kill yourself, suicide methods) †	−0.116	0.649***
Ingin mati (want to die)	0.057	0.153
**Methods**		
Gantung diri (Hanging, hang myself)†	0.420**	0.375*
Minum racun (Drink poison, poisoning)	−0.394	0.459*
Loncat dari (Jumping from)	0.500**	0.078
Obat bunuh diri (Suicide meds, suicide drugs)	0.003	0.549**
Overdosis (Overdose)	0.139	0.218

Note: * indicates *p* < 0.05, ** indicates *p* < 0.01, *** indicates p < .001. † Analyses for individual keywords are repeated from Study 1.

The follow-up analysis revealed that search volumes for low lethality and high lethality methods were significantly correlated (*r* = 0.301, *p* = 0.05). When controlling for low-lethality methods, high-lethality methods were uniquely associated with both suicide rate (*r* = 0.562, *p* < 0.001) and attempt rate (*r* = 0.334, *p* = 0.031). When controlling for high-lethality methods, low-lethality method search volumes were positively associated with attempt rate (*r* = 0.409, *p* = 0.011), but not suicide rate (*r* = *−*0.011, *p* =0.524).

Neither of the loneliness search terms significantly correlated with attempts or deaths (kesepian – suicides: *r* = -0.192; kesepian – attempts: *r* = 0.057; aku kesepian – suicides: *r* = 0.061; aku kesepian – attempts: −0.448). Including these terms in the distress keyword group also did not affect the pattern of findings (distress – suicides: *r* = 0.111; distress – attempts: −0.129).

## Discussion

Across three studies, we investigated the relationship between relative Google Trends search volumes of suicide-related keywords and rates of suicidal behaviours, with the findings suggesting that searches for suicide related keywords having a closer association with suicide attempts and self-harm hospitalisations compared to suicide rate, and that this relationship may be dependent on the type of keyword searched according to the progression from distress, thoughts about suicide, and suicide methods.

In Study 1, we examined the relationship between annual provincial relative search volumes for suicide-related keywords and annual suicide and attempt rates. Our analysis revealed no evidence of a relationship between provincial suicide and attempt rates, consistent with the idea that the fatality rate of suicide attempts differs across provinces. Our analyses also revealed consistently significant relationships between the relative search volumes for suicide-related keywords and attempt rates, but not suicide rates – with only ‘gantung diri’ (hang myself) correlating with suicide rates. This pattern of results is consistent with our hypothesis that internet searches, which reflect suicidality, may be closer in proximity and thus have a stronger association with suicide attempts than to suicide rates.

In Study 2, we investigated the connection between annual relative search volumes for keywords related to suicide and the annual rates for both self-harm hospitalisations and suicide. Our findings did not find a relationship between self-harm hospitalisation and suicide rates, nor between search volumes for any of the keyword categories and suicide rates. However, our results demonstrated significant associations between the search volumes for two keywords and one combination of keywords with self-harm hospitalisation rates. These findings align with our initial hypothesis from Study 1, suggesting that internet searches reflecting feelings of suicidality may be more closely linked to self-harm and suicide attempts rather than to rates of completed suicide.

**Fig. 1:**
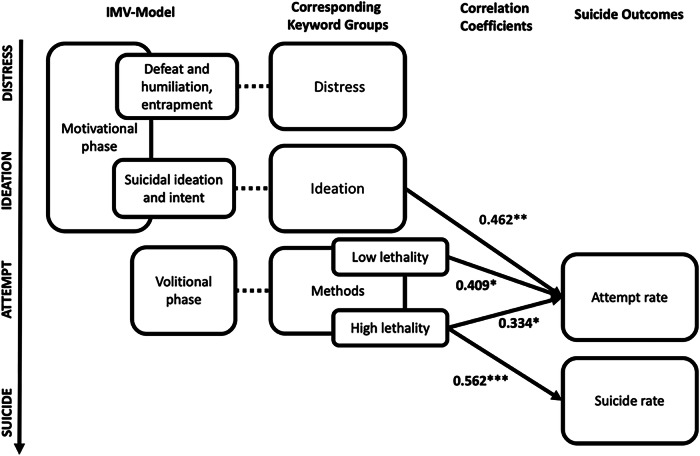
Schematic of the synthesised findings from Study 3. The schematic illustrates how keyword groups were derived from corresponding IMV-Model components and correlated with attempt and suicide rates (* indicates *p* < 0.05, ** indicates *p* < 0.01, *** indicates *p* < 0.001).

In Study 3, we investigated the relationship between categories of keywords, grouped according to which phase they broadly map onto in the IMV-Model, and attempt and suicide rates. In line with our hypothesis, the distress keywords showed no relationship with attempt or suicide rates, and method keywords were uniquely associated with both suicide and attempt rates. Furthermore, when considering the methods keywords based on the method lethality, it appeared that both low and high-lethality keywords had a positive association with attempt rate, but only high-lethality keywords had a positive association with suicide rate. Thus, this suggests that for method-related keywords, the relationship with suicide rate may be driven primarily by high lethality keywords. In sum, the results suggest that keyword categories according to the IMV-Model may differentially predict attempt and suicide rate depending on their proximity to the volitional stage of the IMV-Model or attempt and their lethality – at least in Indonesia. Figure 1 outlines the synthesised findings.

Previous studies have reported conflicting findings on whether Google Trends search volumes are associated with suicide rates. A key paper which strongly argues for the absence of this association was published by Tran and colleagues in 2017^14^. The authors reanalysed several past studies investigating temporal associations between suicide keywords and suicide rates, using best practice time series analyses. While the authors found an association between the relative search volume of ‘suicide methods’ and suicide rates in the US, overall, they did not find an association between ‘suicide’, ‘suicide methods’, and other commonly used keywords, and suicide rates across several countries. The authors concluded that Google Trends data were not reliable in predicting suicide rates. We raise three key points, outlining how our findings and the findings from the study do not contradict one another. First, the authors only examined suicide rates and did not include attempt or self-harm rates. Consistent with their findings, we did not find a relationship between ‘suicide’ and ‘suicide methods’ keywords and suicide rate, as Studies 1 and 2 demonstrated a relationship with attempt/self-harm hospitalisation rates. Secondly, the authors only investigated time series data, whereas we include analyses for single time points across multiple geographic regions. Our time series analysis in Study 2 replicated the finding of a relationship with self-harm but not suicide rate, and also found this association using regional data for a single time period (Studies 1 and 3). Third, our findings suggest that the relationship between relative search volumes and suicide rates depends on the type of keyword, whereas the earlier study did not categorise the keywords into semantic groupings. Thus, our findings support the previous findings, but also provide new insights. Together, these previous and new findings point towards a non-uniform relationship between relative search volumes of suicide-related keywords and suicide and attempt rates, in which not every suicide-related keyword will have a relationship with suicide and attempt rates. However, our study suggests certain relationships reliably emerge across settings and dimensions. Future research needs to carefully consider which keywords to use, informed by what stage of their ideation the individual is likely to be, and whether a temporal or geographic association is being investigated.

Together, these findings cautiously suggest that relative search volume may still be a useful method to monitor suicide outcomes at an aggregate level; however, we must carefully consider what suicide outcomes are of interest, which will inform what keywords are used. Further, the findings from our three studies and two countries suggest that keywords commonly used in past studies^12,14^, that is ‘suicide’ and ‘suicide method’, reliably show an association with attempt and self-harm hospitalisation rather than suicide rate, and are also consistent with previous findings that these keywords were not reliably associated with suicide rate. Future studies may seek to further understand which combination of keywords is best correlated with attempt or suicide rate and validate the keywords using novel data, in addition to replicating our findings across different countries.

Given that a suicide is a fatal attempt, it may be reasonable to assume that suicide attempt and death rates would be correlated. However, we did not find this association. One possibility is that the regional and temporal factors that affect the fatality of an attempt, such as availability of lethal means or accessibility of emergency services, are so diverse and varied across time and regions^17^ that the added statistical variability obscures this relationship. Furthermore, in Study 3, the relative search volume for method-related keywords is correlated to attempt rate, with high lethality keywords also correlated to suicide rate. A previous study found that searching for methods-related keywords corresponded to seriously considering suicide^18^, thus having a relationship with attempt rate. However, the relationship between methods and suicide rate may be driven by relative search volumes of particularly lethal methods. Nonetheless, it is still unclear whether higher searches for more lethal methods reflect higher use of these methods. Future research may seek to investigate whether searches for more lethal methods reflect higher use of these methods.

Linking search terms and potential stages in the progression of suicidal behaviour may help us design better interventions for individuals searching for suicide-related keywords. Our recent work has examined the use of search engine adverts for suicide prevention, in which a person searching for suicide-related keywords is presented with a tailored advert, which when clicked leads to a landing page with resources and help-seeking links^19,20^. One study investigated the effect of using explicit suicide wording in the advert, based on different groups of search keywords including ‘distress’ and ‘ideation’ keywords (called ‘low risk’ and ‘high risk’ respectively in previous study). Using an advert which explicitly mentioned suicide was associated with increased engagement for individuals searching keywords explicitly communicating suicidality^20^ (ideation/high-risk). One possible approach is to tailor these campaigns depending on what keywords the individual uses, indicative of which stage the IMV-Model the individual is likely to be. Tailoring based on the IMV-Model may increase the likelihood that a person is met with relevant and helpful information.

Our studies have several limitations. Firstly, there are concerns regarding data quality and comprehensiveness. Suicides and suicide attempts are known to be underreported in Indonesia due to stigma and data recording issues^21,22^. In Australia, the self-harm hospitalisation data includes only those admitted to a hospital ward, and thus represents only a proportion of self-harm episodes. Nevertheless, this still represents a high-quality and standardised dataset describing self-harm on a national scale. Secondly, the data from Google Trends represents a subset of the total population, in which there may be regional differences in internet accessibility^23^ or search engine preferences. Future studies should examine whether, despite these shortcomings, online engagement is widespread enough to allow for representative data and whether adjustments are needed to account for these variations. Third, there are reservations concerning the utility of relative search volumes. Previous studies have highlighted that data from Google Trends might not be completely aligned with intention in relation to a given behaviour^14^. For instance, individuals may conduct suicide-related searches for reasons unrelated to distress, such as academic research. Further, Google Trends data is normalised, and thus does not provide the raw volume of searches, also preventing calculation of weighted averages for a combination of keywords. Future studies could aim to replicate this analysis using Google Ads data, which offers ad presentation data which can be multiplied by impression share percentage (the proportion of times the ad was shown compared to its eligible presentations) as a proxy for searches, provides higher temporal and geographic resolution, allows grouping keywords, and permits the exclusion of specific keywords to focus on search intent. Another possibility is using Google’s symptom dataset, which provides greater resolution relative to search volumes for health-related keywords but only for Australia, Ireland, New Zealand, the United Kingdom, and the United States. Fourth, we do not yet know how repeated attempts or self-harm influence information-seeking online. For example, an individual may engage in more information seeking prior to an index attempt, compared with subsequent attempts, leading to the possibility of additional confounders in the data and analysis. Fifth, self-harm and suicide attempts are separate outcomes with different underlying methodologies, and thus Studies 1 and 3, and Study 2 are not direct replications. However, of the existing data sources, these are the optimal data sources available to test our hypothesis. Subsequent studies should look to replicating these findings using identical outcomes. Sixth, this study was conducted in only two countries. While other studies typically examine one country at a time, to conclusively determine whether Google Trends data is more closely associated with suicide attempts or self-harm, cross-country comparisons are needed to account for cultural differences. One challenge is that many countries do not record suicide or self-harm and suicide attempt data. To conduct the analyses outlined in our studies, suicide and attempt/self-harm data must be available for the same period of time or the same geographic regions. Future studies should first assess which countries collect both suicide and self-harm/suicide attempt data and for what period or region. Additionally, to ensure that the keywords searched accurately reflect the language and expressions of suicidal thoughts and behaviours in that culture, a co-design process with individuals who have lived or living experience is necessary. In this study, we obtained or had access to suicide and self-harm/suicide attempt data and conducted co-design sessions, either previously or as part of this study, to select the keywords. Seventh, our time series analyses did not investigate different lags in Study 2 as has been done in previous studies. Finally, searches for suicide related keywords may occur across the progression of the IMV model of suicidal behaviour, and not linked specifically to one section over another. However, the concepts in the terms searched may be more closely associated with stages in suicide progression, with previous studies finding that method keywords were found in search histories of individuals who had attempted suicide, suggesting that methods may be searched later in the progression. This study attempts to link this literature with an existing model of suicidal behaviour and future studies should further refine this relationship with models of suicide to best understand the state of mind of the individual searching.

Our findings suggest that the relationship between relative search volumes and attempt and suicide rates may not be a universal one, and keywords may differentially correlate to suicide outcomes according to different stages broadly mapping onto the IMV Model. Future research and efforts should not only replicate these findings across more countries, but carefully consider what outcome (suicide or attempt) is of primary interest, what state the individuals searching are likely to be in, and which keywords to use. Nevertheless, across both geography and time, and in both Indonesia and Australia, the relationship between search volumes of ‘suicide’ and ‘suicide methods’ keywords and attempt rate appears stable. Given the potential for a tool that could monitor changes in attempt and self-harm rates as a macro-level, further exploration is imperative.

## Methods

### Study 1 - Design

We conducted a retrospective, secondary data analysis investigating the relationship between relative search volume for suicide-related keywords across the provinces in Indonesia and attempt and suicide rates. This study was pre-registered on Open Science Framework^24^, with the hypothesis that if suicide rate and suicide attempt rate are positively correlated, then Google search volume would positively correlate with suicide rate through suicide attempt. However, if suicide rate and suicide attempt rate are not positively correlated, then Google search volume would positively correlate with suicide attempt rate but not suicide rate. Given this is secondary data use, Indonesian Psychology Research Ethics through the National Research and Innovation Centre does not require ethics for this study. This study was registered at the University of New South Wales Human Research Ethics Committee portal for secondary data use. Informed consent was not required, given that no individual nor primary data was collected.

### Study 1 - Setting

The study was conducted using data from Indonesia, an archipelago with over 273.8 million people^25^. Data were analysed on a provincial level, the country’s largest sub-national administrative area grouping. Prior to 2022, the country consisted of 34 provinces, which then became 37^26^. Given that the study data in Indonesia was from 2021, we used the 34 provinces in our analysis.

### Study 1 - Google Searches

In line with prior research, we utilised Google Trends^27^ to gather Google search volume data. Google Trends is a freely available online tool that provides a relative search volume index ranging from 0 to 100 for specific terms, rather than raw Google search volumes.

We examined Google Trends data for the year 2021 to investigate the search patterns related to suicide keywords across all 34 Indonesian provinces. Building on prior studies, we selected a set of keywords related to suicide, suicide methods, and painlessness^12^. We also attempted to include a wider range of terms on suicide methods. These terms were translated to Bahasa Indonesia, the national language of Indonesia, and inputted into Google Trends.

The selected suicide keyword was ‘bunuh diri’ (which translates to ‘suicide’ and ‘die by suicide’). The selected suicide methods keywords were ‘cara bunuh diri’ (‘how to suicide’ and ‘suicide methods’). The painlessness keyword was ‘bunuh diri tidak sakit’ (‘painless suicide’ or ‘how to kill myself without pain’). In addition to these keyword categories based on prior work^12^, an additional keyword related to a specific suicide method – ‘gantung diri’ (‘hang myself’) – was added.

Trend data for ‘bunuh diri’ was available for all 34 provinces, while ‘cara bunuh diri’ data was available for 31 provinces, and ‘gantung diri’ data was available for 32 provinces and thus was included in the analysis. However, search volume data for ‘bunuh diri tidak sakit’ was only available for 8 provinces, and thus this term was omitted from subsequent analyses due to limited data availability.

We also averaged the relative search volumes for ‘bunuh diri’ (suicide) and ‘cara bunuh diri’ (suicide methods) across each province, to provide an aggregated metric. While this follows methods of prior work^12^, the aggregated metrics are not directly comparable due to the different data availability for individual terms.

### Study 1 - Suicide and attempt data sources

The data used for suicide and attempt rate were taken from a previous study we conducted to develop Indonesia’s suicide statistical profile. Full information on data preparation is reported elsewhere^21,22^, and is briefly described here.

We obtained province-level suicide attempt and suicide rates for the year 2021. Crude provincial suicide rates were sourced from official police data, which has traditionally served as the authoritative suicide statistics reference^21,22^, given that suicides must be processed within the police system. This data was made available through a collaborative agreement with the Indonesian Ministry of Health and was not open to the public.

Provincial suicide attempt rates were extracted from a publicly accessible report issued by the National Bureau of Statistics (Badan Pusat Statistik)^28^. This report stems from a triannual data collection process known as the Village Potential survey, which covers various domains, including economic, agricultural, and health indicators. The report includes the count of total suicide attempts per province. Further details regarding the survey, its methodology, and major findings are available elsewhere^28^.

### Study 1 - Analysis plan

First, we examined the correlation between annual provincial suicide and attempt rates. Subsequently, we investigated the correlation between search volumes for each of the individual search terms (‘bunuh diri’ [suicide], ‘cara bunuh diri’ [suicide methods], and ‘gantung diri’ [hang myself] and the suicide and attempt rates at the provincial level. The analyses were then repeated using the averaged data from ‘bunuh diri’ and ‘cara bunuh diri’ keywords. Pearson correlation coefficients were calculated in each case for one-way (positive) significance test. All statistical analyses were performed using JASP Statistical Software^29^.

### Study 2 - Methods

In Study 2 we attempted to conceptually replicate the findings of Study 1 by assessing the relationship between relative search volumes, and suicide and self-harm rate data across time in Australia. Whereas Study 1 examined a single year of data at a provincial level, Study 2 examines national-level data across 13 years from 2008 to 2020.

### Study 2 - Design

We conducted a retrospective, secondary data analysis investigating the relationship between hospitalisations for self-harm (as a proxy for attempts), suicide rates, and the volume of searches for suicide-related keywords across multiple years in Australia. This study was registered at the University of New South Wales Human Research Ethics Committee portal for secondary data use. Informed consent was not required given no individual nor primary data was collected.

### Study 2 - Setting

The study was conducted using data from Australia^30^, a nation continent with over 26.4 million people. Data were analysed on a national level.

### Study 2 - Google searches

We extracted national relative search volumes from 2008 to 2020, for the terms ‘suicide’, ‘how to commit suicide’, ‘how to suicide’, ‘how to kill yourself’ ‘painless suicide’, and ‘hang myself’ from Google Trends. Due to a lack of data, Google Trends could not report data for the ‘hang myself’ keyword.

Akin to Study 1, we constructed a combined index by averaging the relative search volumes for all extracted keywords. However, given that in Study 1, the index did not include ‘painless suicide’, we also constructed a second combined index to allow comparison with findings from Study 1.

### Study 2 - Suicide and self-harm hospitalisation data sources

National rates for self-harm hospitalisation and suicide were obtained per year from 2008 to 2020 from the Australian Institute for Health and Welfare website^31,32^.

For hospital-presenting self-harm cases, the Australian Institute for Health and Welfare utilises data from the National Hospital Morbidity Database^32^, which records data on hospitalisations for self-harm with or without suicidal intent. This database does not include individuals who present to community mental health services or general practitioners for self-harm or suicide attempts^31^.

For suicide rates, the Australian Institute for Health and Welfare utilises suicide rates obtained from the Australian Bureau of Statistics. If the coronial process concludes that a suicide has occurred, it is recorded by the Australian Bureau of Statistics^32^.

### Study 2 - Data preparation

Given that correlating temporal data may yield spurious effects, we performed pre-whitening or the Box-Jenkins adjustment on all the yearly relative search volumes, national suicide, and attempt rates as required, following previous studies^14^. An autoregressive integrated moving average (ARIMA) approach was used to examine temporal correlations. For each of the variables, we visually assessed any patterns in the data plot for trends, aiding in the selection of the differencing parameter (d). Following this, correlograms for the autocorrelation function (ACF) and the partial autocorrelation function (PACF) were inspected to inform the candidate model for the autoregressive (AR) and moving average (MA) parameters of the ARIMA model. The candidate model was then compared against similar alternative model specifications using the normalised Bayesian Information Criterion (BIC). To further assess the model adequacy, ACF and PACF correlograms of residuals were inspected to ensure that the residual serial correlations from the selected model specification do not exceed the 95% confidence bands. Once the best-fitting model was identified based on the lowest BIC value and satisfactory residual diagnostics, the residuals were extracted for further analysis. Following that, we conducted cross-correlations on the pre-whitened data, which allowed us to assess the temporal dynamics between the variables.

See Table 4 below for the results of the ARIMA modelling. The only variable that did not require pre-whitening was data for the keyword ‘suicide’.

**Table. 4:** ARIMA Parameters for the variables. (p,d,q) model parameters are auto-regressive model order (p), degree of differencing (d), and moving-average (q) order

Variable	ARIMA Model (p,d,q)
Suicide Rate	0,1,0
Self-Harm Hospitalisation Rate	1,0,0
Keyword: Suicide	0,0,0
Keyword: How to commit suicide	1,1,0
Keyword: How to suicide	1,0,1
Keyword: How to kill yourself	0,1,0
Keyword: Painless suicide	0,1,0
Combined Keywords	0,1,0
Combined Keywords (excluding ‘Painless suicide’)	0,1,0

### Study 2 - Analysis plan

We first investigated the correlation between national suicide and hospitalisation for self-harm rates per year. We then assessed whether the relative search volume for each keyword correlated with the yearly suicide and self-harm hospitalisation rates. Finally, we repeated the analyses combinations of search terms, with and without ‘painless suicide’. Each cross-correlation was performed on the pre-whitened data, and coefficients were obtained at lag 0. For cross-correlation analyses, p-values are not given and thus not used as a threshold for significance; however, significance is determined on whether the cross-correlation coefficient exceeds the 95% confidence interval surrounding a coefficient of 0. All analyses in this study were conducted in SPSS.

### Study 3 - Methods

In Study 3, while overall, suicide-related search volumes from Google Trends have shown a more reliable association with attempts rather than deaths, the Indonesian keyword translating to ‘hang myself’ in Study 1 showed a relationship with both attempts and suicide. ‘Hang myself’ was the only method-specific keyword included, based on the availability of Google Trends data, which in the IMV-Model^16^ would make it more closely associated with the volitional and behavioural phase of the model. Therefore, one possibility is that the association between relative search volume and attempt and suicide rate may depend on what stage the individual is at in the IMV-Model.

We used three keyword groupings broadly based on the IMV-Model. The first “distress” group represented the defeat and humiliation, and entrapment subphase of the IMV-Model, and consisted of keywords surrounding distress and proximal factors. The second “ideation” group represented the suicidal ideation and intent subphase of the motivational phase, and consisted of keywords with explicit suicidal ideation. The third “methods” group focused on the transition between the motivational and the volitional phases, specifically on suicide methods mentioned in the volitional moderators, and consisted of keywords about access and use of suicide methods.

Given that each group reflects a stage of cognition with different proximities to attempt and suicide, we hypothesised that keywords in the distress group would correlate with neither attempt nor suicide rates; keywords in the methods group would correlate with both attempt and suicide rates; and postvention keywords would correlate only with suicide rate. No additional hypothesis was tested for ideation keywords, as these keywords were explored in Studies 1 and 2.

### Study 3 - Design

We conducted a retrospective, secondary data analysis investigating the relationships between relative Google Trends search volumes for suicide-related keywords across the provinces in Indonesia, and attempt and suicide rates. The setting, and suicide and attempt data were identical to Study 1. This study, including keywords, extraction methods, and analyses was pre-registered in the Open Science Framework^24^ Given this is secondary data use, Indonesian Psychology Research Ethics through the National Research and Innovation Centre does not require ethics for this study. This study was registered at the University of New South Wales Human Research Ethics Committee portal for secondary data use. Informed consent was not required given no individual nor primary data was collected.

### Study 3 - Selection of keywords

Given the narrow selection of keywords in Study 1, we aimed to expand this list by including other keywords related to distress and commonly used methods. To achieve this, we developed an advisory group of 11 members, identified through local networks. The group consisted of three clinical psychologists, three psychiatrists, three lived-experience advisors with self-identified lived experience of suicidal thoughts and behaviours or suicide bereavement, and two local suicide prevention experts, including SO from the investigator team. Together, the group discussed local expressions of distress and suicidality in a one-hour remote workshop.

Local expressions of distress related to suicidality commonly revolved around themes of depression, stress, giving up, fatigue, and burnout. The participants agreed that the expressions of suicide and suicide methods remained consistent with those in Study 1. The group suggested terms associated with suicide methods based on their lived experience or clinical encounters as psychologists or psychiatrists. Despite their diverse backgrounds, there was strong agreement among participants, as the language used surrounding suicide is commonly used in clinical practice, popular media and everyday conversation. The group generated a total of 19 keywords: 10 related to distress, 3 to ideation, and 7 to methods. The complete list of keywords can be found in Table 3.

### Study 3 - Google Searches

We examined Google Trends data for the year 2021 to investigate the search patterns related to suicide keywords across all 34 Indonesian provinces. The keywords were selected after consulting an advisory group consisting of clinicians and lived-experience advisors in Indonesia. Table 5 outlines the English keywords and their Indonesian counterparts, indicating which were included in the analysis based on data availability (see Data Preparation, below). Due to language differences, some English keywords may have multiple Indonesian translations and vice versa.

**Table. 5:** Selection of Keywords used in Study 3

Category	English Keyword	Indonesian Keyword	Number of Datapoints	Included
Groups	Distress	-	34	Yes
	Ideation	-	34	Yes
	Methods	-	33	Yes
	Postvention	-	8	No
Distress	Depression	Depresi	34	Yes
		Sedih banget	21	Yes
	Stress	Stress	34	Yes
		Stress banget	2	No
	Giving up	Menyerah	34	Yes
		Menyerah saja	23	Yes
	Fatigue and Burnout	Cape	34	Yes
		Cape banget	6	No
		Lelah	34	Yes
		Aku lelah	33	Yes
Ideation	Suicide and Commit suicide	Bunuh diri	34	Yes
	How to suicide	Cara bunuh diri	31	Yes
	Suicide methods			
	Want to Die	Ingin mati	34	Yes
Methods	Hanging	Gantung diri	32	Yes
	Hang myself			
	Drink poison	Minum racun	24	Yes
	Poisoning			
	Jumping from	Loncat dari	33	Yes
	Suicide meds	Obat bunuh diri	20	Yes
	Suicide drugs			
	Suicide pills	Pil bunuh diri	0	No
	Overdose	Overdosis	31	Yes
Postvention	Suicide reasons	Alasan bunuh diri	8	No
	Why did they suicide	Kenapa mereka bunuh diri	1	No
	Why do people suicide	Kenapa orang bunuh diri	2	No
	Why did my family member suicide	Kenapa keluarga saya bunuh diri	0	No
	Why did my friend suicide	Kenapa teman saya bunuh diri	2	No
	Why did my parent suicide	Kenapa orangtua saya bunuh diri	0	No

Note: Number of datapoints refers to how many of the 34 provinces Google Trends was able to provide data for.

### Study 3 - Data preparation

Similar to Study 1, we also aggregated the individual keywords by averaging the relative search volumes within each group. This yielded relative search volumes for distress, ideation, and methods, and postvention keyword groups. Individual or aggregated keywords were retained for analysis if search volume data was available for 20 or more provinces. Missing data were handled using pairwise deletion method, given that the keywords had been selected on data availability, and listwise deletion would lead to too little data for analysis. Insufficient Google Trends data were available for all postvention keywords. As such, this keyword group was excluded from analyses.

### Study 3 - Analysis plan

The main outcome was to investigate the relationship between the combined search volumes for each keyword category, and attempt and suicide rates. For each keyword group, we investigated whether there was a positive correlation between the search volume and the attempt and suicide rates. If the search volumes for any of these keyword groups were found to correlate with each other, we would assess their relationship with suicide and attempt rate while controlling for the other to encapsulate their unique relationship with the suicide metrics. The secondary outcome was to similarly investigate whether individual keywords were positively associated with attempt and suicide rates, using Pearson correlations testing a one-way positive relationship. All statistical analyses were performed using JASP Statistical Software^29^.

We conducted two follow up analyses. First, the main analysis identified that the methods keyword group showed a relationship with both suicide rate and attempt rate. We therefore investigated whether ‘high lethality methods’ and ‘low lethality methods’ had different relationships with attempts and suicide rate. We combined the keyword search volumes for ‘high lethality methods’ (hanging/‘gantung diri’, jumping from height/‘loncat dari’) and ‘low lethality methods’ (self-poisoning/‘minum racun’, ‘obat bunuh diri’, and ‘overdosis’), and assessed their positive relationships with suicide and attempt rates, while controlling for the other.

Second, given that loneliness is a key factor in suicidal thoughts and behaviours^33^ but was not included as a term in the pre-registration, we repeated the main analysis, adding loneliness to the distress category. These keywords were ‘kesepian’ and ‘aku kesepian’, translated to ‘loneliness’ and ‘I am lonely’ respectively. Each met criteria for inclusion with ‘kesepian’ having 34 data points and ‘aku kesepian’ having 24 data points.

## Data Availability

The Google search datasets generated or analysed during the current study are available on Google Trends. The Australian suicide and self-harm hospitalisation data is accessible through the Australian Institute of Health and Welfare website. The Indonesian suicide and attempt data is not accessible, and was provided under strict requirements by the Indonesian government.
